# Elderberries—A Source of Bioactive Compounds with Antiviral Action

**DOI:** 10.3390/plants11060740

**Published:** 2022-03-10

**Authors:** Mirela Lăcrămioara Mocanu, Sonia Amariei

**Affiliations:** Faculty of Food Engineering, Stefan cel Mare University of Suceava, 720229 Suceava, Romania; sonia@usm.ro

**Keywords:** *Sambucus nigra*, black elder, antioxidant, antiviral, phenolic compounds

## Abstract

In the current context, when more and more unknown pathogens appear, healthy eating and supplementing it with natural products play an increasingly important role in maintaining the health of the body. The European black elder (*Sambucus nigra*), found in abundance in the spontaneous flora, can provide us, as a raw material, elderberries, which have been known for thousands of years as having nutritional and healing properties. The phytotherapeutic principles found in elderberry fruits give them antiviral, antibacterial and antidiabetic properties, antitumor potential, antioxidant, antidepressant and immune boosting properties, as well as a certain impacts on obesity and metabolic dysfunctions. Polyphenols and lectins give elderberry fruits the ability to inhibit coronaviruses, which is a topic of great interest in our times. This article summarizes the existing data regarding the chemical composition, active principles and biopharmaceutical properties of elderberries, as well as their use.

## 1. Introduction

Our life, as we have known so far, has changed radically in the context of the COVID-19 pandemic. Viruses evolve with us, becoming more and more aggressive and pathogenic, resisting more on surfaces, transmitting much more easily to communities. Medical research cannot always keep up with the evolution of these microorganisms; therefore, until the appearance of targeted drugs, in order to keep our body undamaged, it is necessary to eat a healthy diet and supplement it with natural products. Many of these natural products have been known and used for their antiviral properties for thousands of years. Black elder and especially elderberries fall into this category of plants that have, in their structure, bioactive compounds with antiviral action and other actions.

From March 2020 until now, in order to control the COVID-19pandemic, about 300 clinical trials have been conducted in China, of which about 50 are with traditional Chinese remedies and 14 combine these traditional Chinese remedies with traditional Western medicine [1].

Analyses of clinical trials have suggested the effectiveness of specific plant extracts of *Sambucus nigra, Pelargonium sidoides*, or *Cistus incanus* for the treatment of infectious respiratory diseases, regardless of their etiology [2,3,4]. For *Sambucus* and *Pelargonium* polyphenols, antiviral activity against human coronaviruses has been demonstrated in laboratory studies [5,6] so obtaining a dietary supplement containing extracts from elderberries is a topical necessity.

There are more and more current studies on elderberries, and in this review, we have tried to gather as much data as possible about the chemical components of elderberries and how they translate into pharmacotherapeutic effects.

Based on this review, using the data provided by other researchers, we try to obtain a dietary supplement enriched in the most important polyphenols that have antiviral activity.

### 1.1. Origin and History

**Figure 1:**
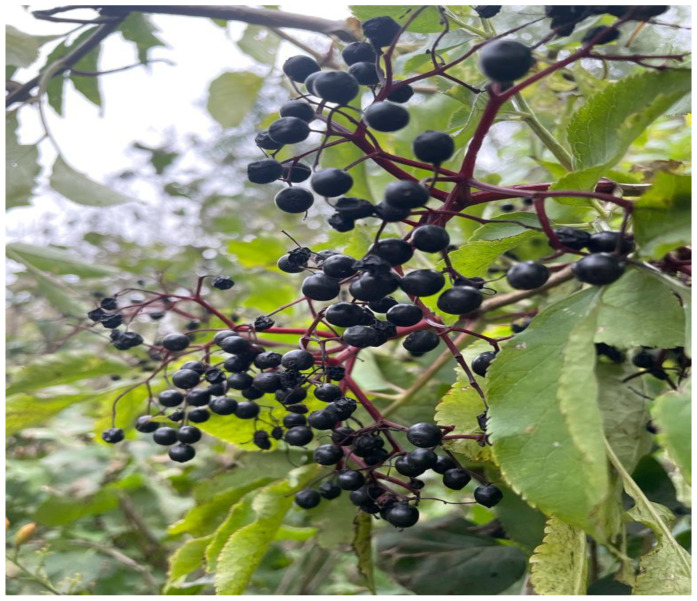
Elderberries found wildly grown.

Described as a medicinal plant since ancient times, the black elder (*Sambucus nigra*) is found in abundance in the spontaneous flora (Figure 1).

Since ancient Egypt, there is evidence of the use of black elder for the treatment of many diseases, in various medicinal formulas, as well as food [7]. Various parts of the plant have been used over the years not only by the population of the Mediterranean basin and surrounding regions [8,9,10] but also by Native Americans [7]. In the 5th century BC, data from Hippocrates, Dioscoridis and Pliny described the use of drugs prepared from *Sambucus nigra* [11].

Germany has the largest production of elderberries in the world, followed by Austria. According to the German Federal Statistical Office, elderberries production amounted to 1576 tons (580 ha) in 2013 and 1759 tons (583 ha) in 2015 [12]. In Austria, the total harvest of elderberries varied between 7.3 and 10.1 thousand tons between 2013 and 2015.

In the past, both the bark and the leaves, flowers, fruits and elder roots were used to make medicines. Although *S. nigra* is not generally considered poisonous, isolated cases of poisoning in animals and man have been reported after eating the bark, leaves, berries, roots and stems [13]. Apart from flowers and ripe fruit, all parts of this shrub contain sambunigrin, a poisonous cyanogenic glycoside, so they are no longer used as recognized remedies, except for those obtained from elder flowers and ripe elderberries. Cyanogenic glycosides can be harmful to animals and humans when they are in excess of 0.5 and 3.5 mg per kg/body. It is also known that it is possible to reduce high levels of cyanide during boiling, fermentation and drying [14].

In the study “The higher the better? Differences in phenolics and cyanogenic glycosides in *Sambucus nigra* leaves, flowers and berries from different altitudes”, the authors investigated the change of phenolics and cyanogenic glycosides in elder leaves, flowers and berries induced by different altitudes and locations. Their conclusions were that elderberries contained the lowest levels of harmful cyanogenic glycosides compared to other analyzed plant parts. Another conclusion of this study is that elderberries and flowers collected at the foothill were characterized by the lowest levels of both beneficial (phenolics) and harmful compounds (CGG) and are suitable for moderate consumption [15].

Elder flowers and fruits are a popular natural source of bioactive compounds, which have a high antioxidant activity. They are used for medicinal purposes and traditionally consumed to prevent or reduce the effects of many diseases, such as respiratory tract infections, pain neuropathy, headache, arteriosclerosis, diabetes, arthritis and cancer [16]. Elderberries and elder flowers are also used in the food industry to produce jams, pies, jellies, ice cream, cakes and other food and alcoholic and non-alcoholic beverages, such as “socata” from elder flower or wine from elderberries.

### 1.2. Botanical Description

*Sambucus nigra*, known as black elder, is part of the *Adoxaceae* family, being a shrub of 3–8 m, with the appearance of a bush, with a wide spread in the spontaneous flora of Europe, North Africa, Asia and America. It includes 20–30 varieties and grows preferably in sunny places, on any type of soil and at various altitudes, still preferring alkaline soils.

In Eastern Europe, the distribution of the plant ends at about 55° E. The altitude limit depends on latitude (e.g., 900 m a.s.l. in the Tatra Mountains and 2200 m a.s.l. in the Atlas Mountains).

Tutin et al. [17] describe this shrub as having arched, brownish-gray branches. The leaves are opposite, imparipinnate, elliptical-lanceolate, with serrated, hairy edges. The flowers are small, white and are united in umbel-shaped inflorescences, each flower having a calyx with five teeth, a corolla with five petals, five stamens with yellow anthers, an ovary and three stigmas. The fruit is a bacciform, globose drupe, up to 6 mm in diameter, black and shiny. Each drupe contains 3–5 seeds inside. Seed ripening is determined by average temperatures. Seeds below 7.2 °C cannot mature [18].

The black elder grows especially in soils rich in nitrogenous bases and rich in phosphates. High levels of phosphate, potassium and mineralizable nitrogen have been observed in a number of soils in *S. nigra* sites [13].

*Sambucus nigra* prefers alkaline soil, however, it is able to grow on another type of soil, even with a pH ranging from 4.2 to 8.02 [19].

### 1.3. Chemical Composition of Elderberries

The chemical composition of *Sambucus nigra* fruit is rich and depends on various factors, such as the variety, location, ripening stage and climatic conditions [16].

The following nutritional and phytotherapeutic principles can be found in 100 g of fresh elderberries (Table 1):

In a meta-analysis published in an article [21], we can find data from existing studies on the chemical composition of elderberries (Table 2):

Tejero et al. [22] show that Sambucus contains a number of organic compounds that probably matter for their medicinal properties. These include simple phenolic acids, including complex polyphenols flavonoids with anthocyanidins and tannins being identified in particular. Major flavonoids present in elderberries are: quercetin-3-O-rutinoside, quercetin-3-O-glucoside, kaempferol-3-O-rutinoside, isorhamnetin-3-O-rutinoside, isorhamnetin-3-O-glucoside and 5-O-caffeylquinic acid. Two flavonol glycosides, Isorhamnetin-3-O-monoglycoside and quercetin-3-O-monoglycoside, have antiulcerogenic activity. Among anthocyanidins, cyanidin-3-O-sambubiozides, cyanidin-3-sambubiozides-5-glucosides, cyanidin-3-O-glucoside and cyanidin-3,5-diglucoside are the most abundant anthocyanins in S. nigra ripe fruit. Cyanidin-3-O-glucoside has been shown to be an anticancer compound which inhibits cell growth [22].

Another special chemical compound found in Sambucus are ribosome-inactivating proteins (RIPs). RIPs are enzymes with N-glycosidase activity on the large RNA of ribosome, preventing it from engaging in protein synthesis [22]. RIPs split adenine 4324 from the 28S rRNA of the large subunit of the rat ribosome or equivalent in other eukaryotes. This adenine is located in a loop that is involved in the interaction of the ribosome with elongation factor 2. As a result, RIPs inhibit ribosomes at the translocation step of translation. In addition, RIPs inactivate ribosomes from certain plants and bacteria. In all these cases, the molecular mechanism of action is the same as in animal cells, namely depurination of ribosomes. Elderberry RIPs also act on DNA and polynucleotides [22].

The most important in terms of active principles are phenolic compounds, which have the most significant role in biopharmaceutical activity presented in Table 3.

### 1.4. Biopharmaceutical Properties of Elderberries

Having in their composition such a great variety of flavones, isoflavones, flavanols, anthocyanins, phenolic acids, lectins and, last but not least, vitamins, elderberries have demonstrated their healing qualities, presenting: antiviral activity [34,35,36,37], potential to stimulate immunity [38,39], antioxidant potential [28,40,41], antibacterial activity [42,43], antitumor potential [44], impact on obesity and metabolic dysfunction [40,45,46,47], antidepressant potential [48,49] and antidiabetic properties [50].

In the current context of the COVID-19 pandemic, the most important biopharmaceutical property of elderberries is the antiviral activity and the potential to stimulate immunity that the different bioactive components of these fruits have.

I cite from Harnett et al.: “Given the body of evidence from preclinical studies demonstrating the antiviral effects of *S. nigra* berry, alongside the results from clinical studies involving influenza viral infections included in this review, pre-clinical research exploring the potential effects of *S. nigra* berry on COVID-19 are encouraged” [51].

First of all, we are talking about flavonoids and polyphenolic components with antiviral, antimicrobial and immune-stimulating effects, proven in numerous in vitro and in vivo studies [35,38,42,43,52,53,54,55].

Then, also in the component of elderberries, we find another extremely important group of components: SNA (*Sambucus nigra* Agglutinin), which are lectins (SNA-IV and SNA-V) and ribosome-inactivating proteins (RIPs).

The elderberry lectins are now referred to as *S. nigra* agglutinins I–V [56].

Ribosome-inactivating proteins (RIPs) and lectins are two types of proteins, which are believed to play a role in the plant defense against phytophagous invertebrates and herbivorous animals. According to a recent definition, lectins are considered as proteins which specifically and reversibly bind carbohydrates without altering their structure. A report on the isolation and partial characterization of SNAV (formerly called nigrin b) demonstrated for the first time that elderberry contains a type-2 RIP [56].

Type-2 RIPs have a more complex structure. They are built up of one, two or four units consisting of two disulphide-bridge-linked, structurally and functionally different polypeptides, called the A and B chains. The A chain possesses N-glycosidase activity and exhibits sequence similarity to the type-1 RIP, whereas the B chain is catalytically inactive but exhibits a carbohydrate-binding activity comparable to that of lectins. Type-2 RIPs are fully capable of agglutinating cells and/or precipitating glycocojugates and, therefore, are also considered as lectins. Since type-2 RIPs possess an enzymic as well as a lectin activity, they are considered as chimaeric proteins [57].

These lectins have been shown not to be toxic in human cell models. In addition, it is very important to remember that, while nonspecific RIPs found in other plants have high toxicity potential, very specific RIPs, which are found in *S. nigra* fruits, have the potential to target specific cells and are therefore candidates that can act as therapeutic agents [22,56,57,58,59,60,61].

In addition to flavonoids, phenolic acids and SNA, *S. nigra* fruits contain peptic polysaccharides, which also appear to play a role in the interaction between *S. nigra* and the human immune system by stimulating macrophages [39,62].

In an analysis of the antiviral activity of several plants, elderberries had activity against several viruses [36].

#### 1.4.1. Influenza Virus

The effects of *S. nigra* fruits against influenza have been the most intensively studied, but research in this field is usually considered only level B, as substantial research is still needed in this field, few mechanisms being explained in terms of inhibition of influenza symptoms [7]. (Assessment level B is in accordance with the validated evidence-based classification standard, equivalent to “You can apply this treatment”: level 2 or 3 studies or level 1 extrapolations).

*Sambucus nigra* flavonoids are able to prevent the virus from entering host cells, preventing the pathogenesis of the flu. Moreover, agglutination of *Sambucus nigra* flavonoids stops influenza infection by competitively inhibiting the virus and subsequently by endocytosis [37].

Another study showed that 5,7,30,40-tetra-O-methylquercetin and 5,7-dihydroxy-4-oxo-2-(3,4,5-trihydroxyphenyl)chroman3-yl-3,4,5-trihydroxycyclohexanecarboxylate have an effect on influenza virus. It was shown that they inhibit H1N1 infection in vitro by binding to H1N1 virions, blocking host cell entry and/or recognition [63].

*S. nigra* fruits appear to prevent influenza infection by competitively inhibiting the influenza virus that binds to host cells to begin its pathogenesis [64,65,66,67,68,69,70,71,72,73].

Studies have also indicated that the effectiveness of elderberries against infection may also be due to immune stimulation [39,62,74].

Other studies have proved that elderberry extract may affect the immune system by enhancing the production of cytokines by monocytes [74]. Torabian et al. [75], in their recent research, indicated that the immunomodulatory property of elderberry extracts manifested by increased expression of IL-6, IL-8, and TNF35.

In order to find the best possible treatments for influenza, more research is needed to determine the extent to which the antiviral effects of *S. nigra* fruit are due to each chemical element of the plant and whether there are synergistic effects between combinations of these constituents to increase their therapeutic potential.

#### 1.4.2. Coronavirus

Antiviral activity against human coronaviruses has also been shown for *Sambucus* polyphenols in laboratory studies [5,6].

*Sambucus nigra* flavonoids have also been proposed as target molecules for SARS-CoV2 therapy in 2020 [76].

In a study published in March 2021, Boroduske et al. [77] presents the results obtained with some species from the northeastern part of Europe: a germplasm with inhibitory capacity against the S protein (spike) SARS-CoV2, the binding of RBD (receptor-binding domain) and hACE2 (angiotensin converting enzyme 2) in vitro. Thus, the best performing *S. nigra* extracts were selected for the evaluation of previously undeclared biological activity-inhibitory capacity against SARS-CoV2.

Concentration-dependent inhibition of RBD binding to S protein ACE2-SARS-CoV2 has been demonstrated in vitro for black elder fruit and flower extracts (IC50 of 1.66 mg DW mL^−1^ and 0.532 mg DW mL^−1^, respectively). The wild elderberries extract showed a higher inhibitory capacity than the “Haschberg” culture elderberries extract which was taken as a reference. This study validates the requirement for bioprospecting the wild germplasm of *S. nigra* and opens directions for further research into new industrial anti-SARS-CoV2 applications of *S. nigra* [77].

#### 1.4.3. Herpes Simplex Virus-Tip 1 (HSV-1)

Kaempferol flavonoid and quercitin from elderberries are cited as having an effect against HSV-1 virus [52].

#### 1.4.4. Helicobacter Pylori

Quercitin stops helicobacter pylori infections. Although low bioavailability contributes to decreasing the in vivo effectivity of natural flavonoids, the current development of novel delivery systems, such as prodrugs, phytosomes and several nanotechnology approaches, enables the inclusion of flavonoids as novel therapeutic tools against *H. pylori* infection [54].

#### 1.4.5. Human Immunodeficiency Virus (HIV)

The flavans were generally more effective than flavones and flavanones in selective inhibition of HIV-1, HIV-2 or SIV infection. Epicatechin from elderberries in particular has been shown to fight HIV. Studies of their effects on the binding of sCD4 and antibody to gp120 indicated that the effective compounds interact irreversibly with gp120 to inactivate virus infectivity and block infection [56].

In a review of five clinical trials involving 936 adults, Harnet et al. [51] show that preparations obtained from elderberries can reduce the duration and severity of respiratory viral infections if given within 48 h of disease onset. The results included in this review come mostly from clinical trials in which participants were given short-term commercial formulations of *S. nigra* fruit for up to 16 days.

The results of the included studies suggest that *S. nigra* fruit preparations (in extract or lozenge) may reduce influenza-like symptoms, including fever, headache, nasal congestion and nasal mucosal secretion in adults, when taken within 48 h of onset of symptoms. In 2–4 days of treatment with *S. nigra*, most adult participants showed a significant reduction in symptoms, averaging 50%. Evidence of the effectiveness of *S. nigra* fruit on cough is currently unclear and inconsistent.

Adverse events were rare, with no serious events reported. Adverse events, reported in two studies, were more common in comparators than in treatments. Currently, there is insufficient scientific evidence to support the use of *S. nigra* in pregnant or lactating women.

### 1.5. Other Biological Activities

#### 1.5.1. Antitumoral Activity

In addition to smoking and UV exposure, lifestyle such as diet, nutrition and physical activities have been shown to play a significant role in many cancers. It is estimated that up to 50% of some cancers can be prevented, many of them through lifestyle and diet changes, with the presence or absence of certain dietary components strongly associated with an increased or low risk of developing cancers [44].

Due to the high content of anthocyanins, the elderberries are cited as having preventive potential, especially in the cancers of the sphere of ENT, esophageal, stomach or colorectal [44]. In addition, anthocyanins play a very important role in cancer prevention because they are antioxidants and prevent angiogenesis, i.e., the formation in cancer cells of capillary vessels providing the oxygen and nutrients needed for small tumors to grow and reproduce [44].

Unfortunately, anthocyanins have poor bioavailability, often only 0.1% of the ingested amount being detectable in urine, but fruit metabolites can be detected in urine, feces, blood and tissues. It is not yet fully understood why anthocyanins have low bioavailability, and thus, this could be seen as a major limitation of the potential efficacy of fruit chemo preventive interventions outside the gastrointestinal tract. However, because about 60% of ingested anthocyanins reach the colon and come into direct contact with the gastrointestinal epithelium, it may explain why antocian-containing berries act as protection against gastrointestinal cancer [44].

Potential anticancer actions in breast or prostate cancer are also cited, but the mechanism of action in these cases has not yet been fully elucidated.

#### 1.5.2. Metabolic Dysfunctions

Another important effect that elderberries have is that of regulating metabolic dysfunctions and, in particular, their use in weight loss due to the content of anthocyanins and proanthocyanins [45].

#### 1.5.3. Other Properties

A study in mice shows that long-term supplementation of the diet with elderberries produces hyperlipidemia but, at the same time, reduces liver inflammation, improving HDL function and thus the stability of atherosclerotic plaque [46].

Recently, the anthocyanin-enriched diet has been shown to increase the antioxidant capacity of plasma [40] and HDL-cholesterol [47]. These pigments are, in fact, incorporated into the membranes and intracellular fluid of vascular endothelial cells, conferring significant effects against oxidative stress in vitro [41].

Anthocyanidins appear to have vasoprotective [78] and neuroprotective effects [48]. The latter is supported by a prospective study, which indicates that fruit and vegetable juices may delay the onset of Alzheimer′s disease [49].

### 1.6. Ways to Use Elderberries

The bunches with ripe berries are cut from the branches, and then, a few hours after harvesting, these fruits must be prepared or preserved in the refrigerator for a maximum of 48 h. Only well-ripened, black-colored, sweet-tasting fruits are collected, starting from the end of August until September, inclusive [79].

In order to keep all the bioactive components in good condition, it was shown in a study [66] that the material (elderberries and flowers) was placed on ice for transport to a laboratory where it was immediately frozen and stored at −80 °C. Frozen plant material was lyophilized for 48 h at −50 °C and 0.04 mbar after final drying for 0.5 h at −65 °C and 0.0054 mbar using an Alpha 1–4LD laboratory plus lyophilizer Martin Christ Gefriertrocknungsanlagen GmbH before extraction.

If not used fresh, elderberries can be dried, but only artificially, using a heat source that ensures a temperature around 40 degrees Celsius. Allow the berries to dehydrate until they resemble raisins. Then, they must be stored in paper bags, in dry and dark places [22].

The following can be prepared from elderberries [79]:

- Fresh juice;
- Tincture;
- Powder;
- Syrup;
- Jams or fermentation wine.

## 2. Conclusions

The European black elder (*Sambucus nigra*), being found in abundance in the spontaneous flora, can provide us, as a raw material, elderberries, which have been known for thousands of years as having nutritional and healing properties.

Polyphenols and lectins give elderberries the ability to inhibit coronaviruses, which is a topic of great interest in our current time.

In addition to flavonoids, phenolic acids and SNA, *S. nigra* fruits contain peptic polysaccharides, which also appear to play a role in the interaction between *S. nigra* and the human immune system.

With this impressive number of bioactive products, these underexploited fruits, fresh or prepared, can become a necessary product in the daily diet of every person.

## Figures and Tables

**Table 1:** Nutritional and phytotherapeutic principles found in 100 g of fresh elderberries.

Phytotherapeutic Principle	Quantity (%) [20]	Quantity (%) [16]
Pectin	0.16	
Total sugars (glucose and fructose)	7.5	7.86–11.5
Total protein		2.7–2.9
Organic acids (citric acid, malic acid, shikimic acid, fumaric acid) Total minerals		1.0–1.3 0.90–1.55
	Quantity (mg)	Quantity (mg)
Potassium	288–305	295–549
Phosphorus		73.5–134
Calcium		57–153
Magnesium		40–74
B2 vitamin	65	
B6 vitamin	0.25	
C vitamin	18–26	
Folic acid	17	
Biotin	1.8	
Beta-carotene	0.36	
Pantothenic acid	0.18	
Nicotinamide	1.48	

**Table 2:** Chemical composition of elderberries.

Anthocyanis	Proanthocyanide/Flavonols	Phenolic Acids	Organic Acids	Vitamins	Sugars	Lectine
Cyanidin-3-sambubioside, Chrysanthemin, Cyanidin-3,5-diglucoside, Cyanidin-3-sambubioside-5-glucoside, Antirrhinin, Callistephin, Tulipanin, Pelargonidin-3-sambubioside, Peonidin-3-glucoside, Peonidin-3-sambubioside, Peonidin monoglucuronide, Chrysanthemin monoglucuronide	Epicatechin, Rutin, Isoquercetin, Kaempferol-3-O-rutinoside, Isorhamnetin-3-O-rutinoside, Quercetin, Astragalin, 5,7,3′,4′-tetra-O-methylquercetin, unidentified quercetin acetylhexosides, unidentified quercetin hexoside pentosides	caffeoylquinic acid, feruloylquinic acid, coumaroylquinic acid	shikimic acid, fumaric acid, citric acid, malic acid, tartric acid, valeric acid	ascorbic acid, retinol	fructose, sucrose	SNA-IV (*Sambucus nigra* agglutinin-IV), SNA-V (*Sambucus nigra* agglutinin-V)

**Table 3:** The content of phenolic compounds in elder fruits and flowers.

Compound	Content in Fruit	Content in Flowers	Source
**Total polyphenolics**			[23,24,25,26]
mg GAE/100 g FW	364–582	1021.7	
mg GAE/100 g DW extract	4917–8974		
mg CAE/100 g FW	2684–4480	3702–5333	
mg CE/100 g FW	622–672		
**3-caffeoylquinicacid** (neochlorogenic acid)			[23,25,27,28]
mg ChAE/100 g FW	0.7–4.4		
mg/kg FW		510.6	
mg ChAE/g DW	0.05–0.40		
mg/g DW		0.8–2.4	
**4-caffeoylquinicacid** (cryptochlorogenic acid)			[23,25,28]
mg ChAE/100 g FW	1.2–2.5		
mg/kg FW		31.4	
mg/g DW		0.6–1.5	
**5-caffeoylquinicacid** (chlorogenic acid)			[23,27,28]
mg ChAE/100 g FW	26.4–35.9		
mg ChAE/g DW	0.53–1.22		
mg/g DW		10.1–20.7	
**Kaempferol-3-rutinoside**			[23,25,28]
mg rutin/100 g FW	0.7–1.2	0.64	
mg/g DW		0.2–3.0	
**Kaempferol-3-glucoside**			[25,29]
g/100 g extract	1.05–1.79	1.28–2.50	
mg/kg FW		3.5	
**Quercetin**			[30,31]
mg CGE/100 g FW	2.7–4.5		
mg/100 g FW	29–60		
**Quercetin-3-rutinoside (rutin)**			[23,25,27,28,29,31,32]
mg rutin/100 g FW	42.6–95.6		
mg rutin/g DW	6–14	11.6–42.3	
g/100 g extract	10.86–15.39	132.69–202.08	
mg CGE/100 g FW	35.59–52.02		
mg/kg FW		3265.1	
**Quercetin-3-glucoside** (isoquercitrin)			[23,24,26,27,28,29]
mg rutin/100 g FW	3.9–14.9		
g/100 g extract	1.79–3.01		
mg CGE/100 g FW	6.4–26.5	5.37–9.67	
mg rutin/g DW	0.11–1.08	0.4–1.9	
mg/kg FW		20.2	
**Isorhamnetin-3-rutinoside**			[23,25,28]
mg rutin/100 g FW	0.3–2.2		
mg/kg FW		888.0	
mg/g DW		2.0–7.5	
**Isorhamnetin-3-glucoside**			[23,25,28]
mg rutin/100 g FW	0.1–0.3		
mg/kg FW		61.6	
mg/g DW		0.2–1.0	
**Catechin**			[25]
mg/kg FW	10.7	6.8	
**Epicatechin**			[25]
mg/kg FW	81.3	254.3	
**Total anthocyanins**			[23,24,27,29,30,31,32,33]
mg CGE/100 g FW	170–343		
	518–1028		
	664–1816		
	602.9–1265.3		
mg CGE/100 g DW	408.6–1066.6		
	39–153		
mg CGE/100 g extract	285–1326		
mg CSE/g DW	8.33–101.40		
g CSE/kg FW	1.9–20.2		
g/100 g extract	48.46–52.89		
mg/100 g FW	254–841		
**Cyanidin-3-sambubioside-5-glucoside**			[23,27,30,31]
mg CGE/100 g FW	16.0–59.2		
	14–47		
	19.52–53.49		
mg CSE/g DW	0.86–11.50		
**Cyanidin-3,5-diglucoside**			[23,27,30,31]
mg CGE/100 g FW	8.2–19.5		
	5–36		
	7.41–23.29		
mg CSE/g DW	0.12–5.22		
**Cyanidin-3-sambubioside**			[23,27,30]
mg CGE/100 g FW	122.2–269.1		
	269–656		
mg CSE/g DW	270.8–630.8		
**Cyanidin-3-glucoside**			[23,24,27,29,30,32]
mg CGE/100 g FW	204.6–481.4		
	361–1266		
	221.4–586.4		
mg CSE/g DW	2.74–49.50		
g/100g extract	21.41–25.18		
mg CGE/g DW	14–78		
**Cyanidin-3-rutinoside**			[23]
mg CGE/100 g FW	1.49–9.63		

Abbreviations: GAE—gallic acid equivalents, CAE—caffeic acid equivalents, ChAE—chlorogenic acid equivalents, CGE—cyanidin-3-glucoside equivalents, CSE—cyanidin-3-sambubioside equivalents, DW—dry weight, FW—fresh weight.

## Data Availability

Not applicable.
